# Towards stem cell therapies for skeletal muscle repair

**DOI:** 10.1038/s41536-020-0094-3

**Published:** 2020-05-11

**Authors:** Robert N. Judson, Fabio M. V. Rossi

**Affiliations:** 1 STEMCELL Technologies Inc, Vancouver, BC Canada; 2 Biomedical Research Centre, Department of Medical Genetics, University of British Columbia, 2222 Health Sciences Mall, Vancouver, BC Canada

**Keywords:** Muscle stem cells, Cell delivery

## Abstract

Skeletal muscle is an ideal target for cell therapy. The use of its potent stem cell population in the form of autologous intramuscular transplantation represents a tantalizing strategy to slow the progression of congenital muscle diseases (such as Duchenne Muscular Dystrophy) or regenerate injured tissue following trauma. The syncytial nature of skeletal muscle uniquely permits the engraftment of stem/progenitor cells to contribute to new myonuclei and restore the expression of genes mutated in myopathies. Historically however, the implementation of this approach has been significantly limited by the inability to expand undifferentiated muscle stem cells (MuSCs) in culture whilst maintaining transplantation potential. This is crucial, as MuSC expansion and/or genetic manipulation is likely necessary for therapeutic applications. In this article, we review recent studies that have provided a number of important breakthroughs to tackle this problem. Progress towards this goal has been achieved by exploiting biochemical, biophysical and developmental paradigms to construct innovative in vitro strategies that are guiding stem cell therapies for muscle repair towards the clinic.

## Therapeutic potential of skeletal muscle stem cells

Unlike other adult muscle tissues, such as the heart, skeletal muscle is endowed with an excellent regenerative capacity due to a population of tissue-resident stem cells. Satellite cells are positioned between the basal lamina and sarcolemma of myofibers and comprise less than 5% of mononucleated cells in the tissue. Generally quiescent, MuSCs are characterized by the expression of transcription factor Pax7, which is indispensable for their self-renewal^1,2^. In response to tissue injury, MuSCs become activated, enter the cell cycle and progress along a stepwise process to generate proliferating MyoD positive progenitors (myoblasts) before differentiating and fusing to repair damaged myofibers. MuSCs, or at least a subset of MuSCs^3–5^, are also capable of extensive self-renewal, allowing skeletal muscle to regenerate after repeated rounds of injury. In vivo cell labeling studies have shown MuSCs can be isolated and transplanted into donor muscle, resulting in robust engraftment into myofibers and the satellite cell niche^3^. These animal models of autologous stem cell therapy have demonstrated that transplantation of as little as 900 MuSCs/tibialis anterior (TA)^6^ (20–200 MuSCs/mg muscle) can improve muscle function in mouse models (mdx mice) of Duchenne muscular dystrophy (DMD) and sarcopenia (22–24-month-old mice), providing clear pre-clinical evidence of efficacy^7^. However, early-phase clinical trials in DMD patients showed that transplantation of MuSCs and specifically, of their in vitro progeny, myoblasts, was not successful at restoring sufficient dystrophin expression, resulting in negligible strength recovery outcomes^8,9^. Follow-up studies demonstrated injected cells displayed poor survival and limited capacity migrate into donor tissue^10^. The underlying basis of these failings came from the realization that although freshly isolated MuSCs can engraft and self-renew in situ, providing a source of new myonuclei, this capacity is rapidly lost when MuSCs are cultured, preventing the in vitro expansion required to generate a sufficient number of cells for a clinically beneficial effect following transplantation^11^ (doi:3.0.co;2-8">10.1002/(sici)1097-4598(199607)19:7<853::aid-mus7>3.0.co;2-8.). Renewed efforts to overcome this obstacle have led to the development of culture platforms that mirror an in vivo microenvironment supporting stem cell self-renewal and can be utilized for the cultivation of MuSCs that maintain regenerative capacity.

## Pharmacological modulators of myogenic cell fate

Traditional culturing methods for MuSCs are not permissive for long-term cell expansion and instead lead to loss of Pax7 expression, spontaneous differentiation, senescence and a consequent loss of regenerative potential^11^. Supplementation of culture media with small molecules that target signaling pathways controlling myogenic differentiation has therefore been utilized to maintain MuSCs in an immature state and enhance their ability to self-renew after engraftment. Inhibition of p38 mitogen-activated protein kinase (p38 MAPK) with small molecules (such as SB202190, SB203580, SB239063 and SB706504) has been shown to be a particularly effective approach^7,12,13^. Indeed, treatment of cultured murine or human MuSCs with SB molecules was sufficient to rescue the proliferative potential of aged MuSCs and restore their regenerative potential following transplantation^7,12,13^. This treatment dramatically enhanced the self-renewal capacity of engrafted MuSCs in serial transplantation experiments and led to a restoration in muscle strength in aged mice^7^. Work from our own lab also supports such methods. Methyltransferase Setd7 is a potent regulator of MuSC differentiation and can be inhibited with small molecule (R)-PFI-2^14^ during in vitro expansion to boost proliferation of murine and human MuSCs^15^. Propagation with (R)-PFI-2 enhanced the therapeutic potential of MuSCs by restoring muscle strength in mdx mice after transplantation. Similarly, detailed studies by the Crist lab^16,17^ have demonstrated the translational machinery of MuSCs can be targeted with chemical molecules to inhibit the production of pro-differentiation factors. Inhibitors of eIF2alpha phosphatase Gadd34/PP1 such as Sal003^17^ as well as a more potent analog, ‘C10’^16^, were shown to be particularly effective. Treatment of mouse MuSCs with 5 µM C10 in culture media delayed activation of the myogenic program, boosted the expression of Pax7 and maintained the engraftment potential of MuSCs to levels comparable to the transplantation of freshly isolated cells. Interestingly, C10 also permitted the expansion of MuSCs over multiple passages, generating up to 13 fold more Pax7+ MuSCs compared with standard culture conditions^16^. Together these studies demonstrate the utility of pharmacological inhibitors of myogenic differentiation to enhance propagation of MuSCs ex vivo and boosting transplantation potential.

Additional approaches have included elucidating factors that regulate the initiation of cell fate decisions in MuSCs. This has led to the identification of the Wnt7a/Fzd7/Vangl2 and EGFR/Aurka pathways as key regulators of the balance between symmetric and asymmetric MuSC divisions^18,19^. Wnt7a signaling skews MuSCs towards symmetric divisions^18^ (increasing the pool of self-renewing stem cells), whereas activation of EGFR signaling promotes asymmetric divisions^19^ (increasing the pool of committed progenitors). Wnt7a has been shown to increase the motility of MuSCs, resulting in enhanced engraftment efficiency and functional improvements in muscle strength when transplanted into mdx mice^20^. Mechanistically, Wnt7a and EGFR act in a spatiotemporal manner to control MuSC mitosis in the myofiber niche by modulating centrosome recruitment and thus the apicobasal orientation of MuSC divisions^18,19,21^. Spatial and temporal modulation of these pathways in vitro has yet to be achieved. However, such a strategy, balancing the symmetry of cell divisions between commitment and self-renewal, could provide a powerful mechanism to guide the cell fate of MuSCs during ex vivo expansion or even following transplantation for therapeutic purposes.

## Engineering biophysical cues ex vivo

Biophysical properties such as substrate stiffness, geometry, and extracellular matrix (EMC) composition have directed the engineering of culture systems that attempt to mimic the architecture of the MuSC niche. Unlike rigid plastic cell culture dishes, growing MuSCs on soft hydrogels^7,22^ that resemble the elastic modulus of muscle tissue (≅12 kPa) supports in vitro self-renewal. MuSCs seeded onto soft hydrogels maintain engraftment and niche repopulation capacity. A recent study by Quarta et al.^23^ described the development of engineered muscle fibers (EMFs): an artificial culture platform modeled on the native biophysical features of myofibers. EMFs replicated several physical cues of muscle tissue in geometry, elastic modulus, ECM composition, and structural organization. This system was utilized to preserve MuSC quiescence, allowing a window for genetic manipulation (e.g., gene correction) of cells ex vivo whilst maintaining self-renewal potential following transplantation. The ECM is also an integral component of the MuSC niche and is composed of a mixture of collagens^24^, fibronectins^21^, and laminins^25^, contributing to MuSC maintenance^26^. Mimicking specific ECM niche composition in vitro has also been shown as an effective strategy to help the expansion of immature MuSCs. For example, Ishii et al.^27^ recently identified specific Laminin isoforms as a key component of the MuSC niche. A culture platform including recombinant Laminin-E8 molecules supported expansion of murine and human MuSCs with enhanced engraftment potential^27^.

## Biomaterial technologies for muscle repair

Bioengineering approaches have been employed to boost the delivery efficiency of MuSCs following expansion, which remains an additional and significant translational challenge^28^. Injured, aging and dystrophic tissue possess a harsh inflammatory microenvironment and impaired structural integrity that is not conducive to efficient survival and cell engraftment following transplantation^28,29^. Progress towards tackling this obstacle has been made with the generation of injectable, encapsulating, 3D biomaterials that mirror the structural architecture of muscle and can be loaded with a cocktail of growth factors to enhance transplantation efficiency of MuSCs. Examples have included synthetic macromers^30^, bioactive hydrogels^31–34^ and other biomimetic scaffolds^35^ (reviewed by Wolf et al.^36^) An impressive illustration of this approach was by Han et al.^30^ who through a series of iterative design optimization steps, recently engineered a fully synthetic, degradable, poly(ethylene glycol) (PEG)-4MAL hydrogel functionalized with integrin-binding Arg-Gly-Asp (RGD) peptides. When co-injected with MuSCs, this biomaterial resulted in boosted MuSC proliferation, survival and in vivo engraftment into dystrophic and aged mouse skeletal muscle. Building on this strategy, Sleep et al.^35^ developed a liquid crystalline scaffold containing nanofibres which facilitate the spatial alignment of MuSCs following encapsulation and engraftment into tissue. This system also permitted loading of recombinant growth factors, enhancing engraftment efficiency of cells even in the absence of an injury stimulus, which is often used to yield a supportive environment for the exogenous cells^35^. Additional examples of growth factors used to load biomatrices to enhance transplantation efficiency of myogenic cells have included IGF-1, VEGFR^37,38^ and Wnt7^39^.

Historically poor clinical outcomes associated with volumetric muscle loss (VML)^40,41^ have also provided a major driving force for developing biomaterial technologies. Unlike acute experimental injury models (cardiotoxin) or transgenic models of human myopathies (MDX mice), VML is characterized by a permanent loss of muscle tissue and is often due to trauma such as, for example, battlefield injuries^42^. VML lesions overwhelm endogenous muscle repair mechanisms such as that MuSCs are unable to restore tissue structure, resulting in chronic functional deficits. Researchers have attempted to utilize biomaterials to provide a guide or inductive template to recruit endogenous stem/progenitor cells to the sites of VLM and trigger repair. Examples of this approach have included the transplantation of acellular scaffolds constructed of synthetic or bioactive ECM materials into VML lesions^36^. Although similar biomaterial scaffolds have been demonstrated to improve afflictions in some tissues^43^, reports on the effectiveness of this strategy to induce meaningful recovery of muscle function have been highly variable^44–49^. The basis of these failings is underscored by observations of limited infiltration of endogenous MuSCs into scaffolds, negligible de novo muscle fiber formation and extensive fibrosis^45^. However, a recent iteration of this approach has been to perfuse biomaterial or ECM scaffolds with multiple cell populations resident in muscle, including MuSCs, prior to transplantation providing a cellular source not just for new muscle fiber formation, but also for vascularization and matrix deposition^31,50–53^. This strategy has provided encouraging initial results with implanted bioconstructs facilitating widespread de novo muscle fiber formation, evidence of new vessel networks and seemingly significantly less fibrotic accumulation than acellular scaffolds. Importantly, evidence also indicates treatment of VML with such cellularized ECM/scaffolds can lead to a partial restoration of muscle function, assessed by isometric torque force measurements^31,53^.

Together, it is clear that continued progress in the development of biomaterial technologies will surely be critical in mitigating the inherent limitations and challenges presented by direct cell delivery and improving therapeutic outcomes for muscle diseases and injuries.

## Mimicking the inflammatory milieu

Mirroring the complex inflammatory microenvironment found within regenerating muscle during the in vivo expansion of MuSC has been proposed as a potential strategy to sustain MuSC expansion in culture, without loss of potency^54,55^. In this vein, Fu et al.^54^ discovered CD3 + T-cells accumulate in skeletal muscle 3–5 days post injury (a time of rapid expansion of undifferentiated MuSCs) and that co-culturing CD3 + T-cells with MuSCs facilitates large-scale expansion of MuSCs over 20+ passages. Analysis of the T-cell secretome from condition media revealed that a combination of just four inflammatory cytokines (IL-1alpha, IL-13, TNF-alpha and INF-gamma) added to MuSC culture media was sufficient to promote long-term expansion of MuSCs. Remarkably, these culture conditions allowed MuSCs to preserve phenotypic characteristics of freshly isolated MuSCs throughout long-term expansion, as well as enhanced engraftment potential into irradiated muscle and effective homing of transplanted MuSCs to the stem cell niche. Similar observations have been made with inflammatory mediator Prostaglandin E2 (PGE2), which is dramatically elevated in muscle tissue following injury and whose modulation in vivo could also affect MuSC stemness^55^. An acute 24 h treatment regime of MuSCs with PGE2 immediately following isolation induced >6-fold increase in total cell yield after 7 days in culture. Mechanistically, PGE2 was shown to act through the EP4 receptor, providing downstream regulation of cell proliferation via transcription factor Nurr1. Co-injection of MuSCs with PGE-2 also enhanced the regenerative capacity of MuSCs, improving myofiber engraftment and stem cell repopulation. These and similar studies reveal that the inflammatory milieu of regenerating muscle is critical for proper MuSC function, and it provides a promising blueprint to design in vitro strategies to expand MuSCs with therapeutic potential.

## Pluripotent stem cells as a source of myogenic progenitors

Human pluripotent stem cells (hPSCs) generated from the reprogramming of somatic cells possess the ability of indefinite self-renewal and can be patterned to differentiate into daughter cells that give rise to nearly all cell types of the body. Unlike MuSCs isolated from muscle tissue, PSCs possess the ability of indefinite self-renewal and could, therefore, provide a plentiful source of cells for therapeutic applications.

Unlike cell types from the neuroectoderm, cardiac and endoderm lineages, efficient differentiation of hPSCs into skeletal muscle cells has proved challenging^56^. Both transgene-mediated and soluble factor-directed differentiation protocols have been described. Initial attempts have been based on ectopic expression of myogenic regulatory factors, such as PAX3^57^, PAX7^57,58^ and MYOD1^59,60^ via viral gene delivery. These reprogramming approaches have been reported to produce myogenic cells at high efficiency with in vitro and in vivo differentiation potential. However, how well these cells reflect myogenic progenitors, which in vivo rely on tightly controlled spatial and temporal cues to transition through distinct developmental cellular intermediates, remains to be seen^61^. Random integration of viral DNA risks jeopardizing disease modeling by masking novel pathophysiological features and is also not as well suited to the tight regulatory landscape of cell therapy^62^.

More recent efforts have focused on optimizing directed differentiation protocols - that is, modulating relevant signaling pathways in PSCs via small molecules, growth factors and cytokines to produce skeletal muscle cells by recapitulating the developmental cues of embryogenesis^63^. The most established of these protocols was developed by the Pourquie lab^64–66^ and emerged based on mapping expression patterns from precursors of myogenesis during mouse development. This protocol produces a confluent heterogenous monolayer of myogenic cells after 30–50 days, that can be enriched for expandable PAX7 + myogenic progenitors by enzymatic dissociation and sub-culturing. Numerous additional directed differentiation protocols have now also been described (Reviewed in Chal et al.^63^) sharing similar features in their cadence and typically producing mixed cultures of PAX7 + progenitors, MYHC + myofibers and other cells after 15–50 days, with varying efficiencies. Considerable effort has also gone into identifying cell surface markers than can be used to enrich PAX7 + hPSC derived myogenic progenitors with MuSC characteristics, including in vivo engraftment potential. Hicks et al.^67^ recently reported that using a directed differentiation protocol^68^, highly potent myogenic cells are enriched in an ERBB3^+^NGFR^+^ cellular fraction. When isolated by FACS, ERBB3^+^NGFR^+^ cells possessed enhanced myotube formation in vitro and could engraft into muscle of mdx-NSG mice at a similar efficiency to uncultured fetal myogenic progenitors, restoring dystrophin expression in 10–15% of myofibers. In support of these findings, NGFR has been shown to be enriched in ES-derived PAX7:eGFP+ cells and was used to isolate progenitors with enhanced myogencity^69^. Similarly, using a PAX7/MYF5 double reporter hPSC line Wu et al.^70^ described an optimized 15-day differentiation protocol to generate expandable hPSC derived myogenic progenitors via enrichment of CD24^neg^/CD10^+^ cells. These cells possessed enhanced myotube formation and robust in vivo engraftment, generating h-dystrophin+ myofibers when transplanted into mdx/nsg mice. The identity and effectiveness of such markers to enrich for hPSC derived myogenic progenitors may be dependent on the timing and parameters of the differentiation protocol being employed and add a potentially challenging layer of complexity in our understanding of such approaches. Nevertheless, utilizing these protocols can generate large numbers^71^ of expandable myogenic progenitors. In vivo transplantation experiments in mice have provided proof-of-principle evidence of the therapeutic potential of PSC-derived myogenic progenitors, which can engraft into injured skeletal muscle tissue and restore muscle function in disease models^72^.

## Challenges and opportunities ahead

Several hurdles still face the translation of muscle stem cell therapies towards the clinic. First, our understanding of the precise molecular underpinnings that differentiate self-renewal competent, efficiently engrafting MuSCs from progenitors that can only engraft myofibers remains insufficient. We expect more work in this area to provide important breakthroughs about the mechanisms controlling the regenerative potential of MuSCs. Second, culture systems have been optimized primarily using mouse MuSCs, and whether these same methods can be applied to human MuSCs needs confirmation - especially as murine and human MuSCs have distinct functional characteristics^73^. Third, transition towards the clinic will eventually require reagents that are better compliant with regulations, such as serum-free media and chemically defined culture systems. Substituting components such as animal sera is not trivial and represents a key biological obstacle to generating clinical-grade cells under current Good Manufacturing Practices (cGMP). Finally, experience from recently approved CAR-T therapies clearly demonstrates that personalized medicine approaches can be incredibly expensive due to inherent fixed costs of generating patient-specific cells. Questions of affordability and projected efficacy of cell therapy strategies should be considered against the backdrop of competing state-of-the-art advances in, for example, AAV-mediated gene therapies and CRSPR-based gene correction approaches for muscular dystrophies, which have also shown tremendous promise in correcting genetic defects in pre-clinical myopathy models and are entering the clinic^74–78^.

Cell therapy strategies may have advantages in the treatment of afflictions without a simple monogenic etiology or to repair muscle tissue damaged in severe trauma such as VML. Indeed, VML has emerged as likely the best candidate for utilizing a cellular therapy approach as the pathobiology necessitates the de novo formation of muscle fibers and total reconstruction of tissue architecture for its treatment. Combining cellular therapy and tissue engineering has provided some encouraging results in animal models. The deployment of these strategies will depend on further optimizing cellular bioconstructs capable of restoration of mature muscle tissue, vascularization, organized ECM composition and critically, innervation. Recent progress has begun defining, in addition to MuSCs, which myogenic cell types are important for facilitating muscle repair. These studies have highlighted a supporting role for endothelial cells, fibro/adipogenic progenitors and pericytes as well as developing novel strategies to promote innervation of bioconstructs through exercise^31^. However, more advancements are still required, especially to overcome the lack of innervation, for which we envision solutions to promote repair of peripheral nerves and NMJ formation will hold the key.

Recent advancements in protocols to differentiate hPSCs into myogenic progenitors represent a major opportunity to overcome the obstacles of MuSC expansion. In addition to the progress mentioned above, human PSCs have also been engineered that lack the machinery for antigen processing/presentation, generating hypoimmunogenic cells capable of evading the host immune system following transplantation^79,80^. Such advances could pave the way to the creation of “universal donor cells”, simultaneously circumventing issues related to immune-rejection and abolishing the fixed costs of generating patient-specific cells for therapy. Although challenges remain to address the long-term malignant potential of such immune evasive cells following transplantation^81^. A degree of ditocomy exists on the relative merits of directed vs transgene-mediated differentiation methods to generate myogenic progenitors. To date, it appears transgene approaches may be more efficient^72^. However, their compatibility with the strict regulatory landscape of cell therapy is unknown – and indeed it is telling that the most clinically advanced cell therapies involving hPSCs (e.g., differentiation into RPE cells^82^, cardiomyocyte progenitors^83^ and insulin-secreting beta-like cells^84^) have all employed directed differentiation methods. That said, several problems also exist with directed differentiation approaches. First, there is considerable inconsistency in the timing and targeting of specific signaling pathways described in these protocols. For example, manipulating Wnt, TGF-beta, Notch, BMP, PI3K, Hedgehog and Retinoic Acid pathways have all been implicated in various combinations to pattern PSCs into skeletal muscle^56,63^. This makes it impossible to infer whether cells generated across distinct protocols are phenotypically equivalent. Second, the majority of methods are based on patterning of embryoid bodies which pose established challenges with consistency in directed differentiation methods. Third, some protocols rely on FACS purification to enrich mesodermal/myogenic cells^67,85–88^ or the use of conditioned media^71,87^, making these methods laborious and eventually, too expensive. Finally, very few directed differentiation protocols described to date have demonstrated in vivo engraftment potential that leads to a functional improvement of skeletal muscle in the setting of disease. Together these represent key challenges that remain to be overcome in order to move these cells towards clinical applications.

**Fig. 1:**
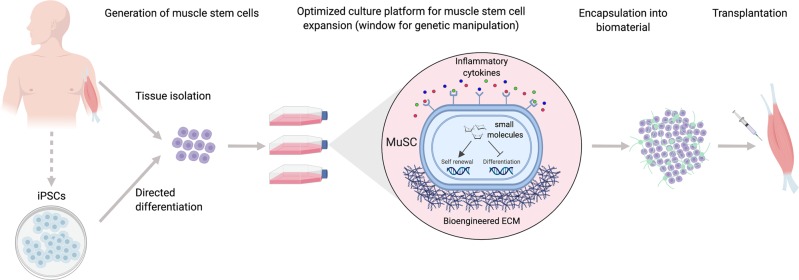
Therapeutic workflow for muscle stem cells in regenerative medicine utilizing multiple convergent advances. **(Left)** Muscle stem cells (MuSCs) prospectively isolated from muscle tissue or derived from differentiation of induced pluripotent stem cells (iPSCs). **(Middle)** Ex vivo expansion of MuSCs in optimized culture platform that mimics in vivo microenvironment supporting self-renewal. **(Right)** Delivery of undifferentiated MuSCs into diseased/damaged muscle with biometric scaffold boosting transplantation efficiency.

Although discourse does exist about the optimal protocol to generate PSC-derived myogenic progenitors, this method has a number of advantages over a tissue-derived autologous cell transplantation strategy and provides an exciting therapeutic avenue for exploration. Methods to preserve the self-renewal potential of MuSC in vitro will still be needed independent of how such MuSC are obtained. In summary, although significant and daunting hurdles remain, we believe the deployment of PSC-derivation protocols in synergy with the innovative biochemical and biophysical cell culture systems described above could greatly accelerate therapeutic development, overcoming the historical failings of previous methods and bringing stem cell therapies for muscle repair a step closer to reality (Fig. 1).
